# Potential Impact of Direct Versus Indirect Central Venoarterial Extracorporeal Membrane Oxygenation (VA-ECMO) Cannulation in Refractory Postcardiotomy Cardiogenic Shock

**DOI:** 10.7759/cureus.69415

**Published:** 2024-09-14

**Authors:** Wael Al Ghareeb, Mohammad Aldabbas, Abdou Sheikh Ali, Baravan Al-Kassou, Christopher Gestrich, Georg Nickenig, Oliver Dewald, Fritz Mellert

**Affiliations:** 1 Department of Cardiac Surgery, European Medical School Oldenburg-Groningen, Carl von Ossietzky University of Oldenburg, Oldenburg, DEU; 2 Department of Medicine II, Heart Center Bonn, University Hospital Bonn, Bonn, DEU; 3 Department of Cardiac Surgery, University Hospital Erlangen, Friedrich-Alexander-University, Erlangen, DEU

**Keywords:** cardiac surgery, central cannulation, postcardiotomy shock, survival outcomes, veno-arterial extracorporeal membrane oxygenation (va ecmo)

## Abstract

Background

Central venoarterial extracorporeal membrane oxygenation (VA-ECMO) is a commonly employed strategy to support patients in refractory postcardiotomy cardiogenic shock (RPCS). This support can be provided using either indirect central ECMO (icECMO) with a closed thorax or direct central ECMO (dcECMO) with an open thorax.

Methods

This single-center retrospective analysis included 60 patients undergoing central VA-ECMO for RPCS from January 2019 to December 2020. The primary endpoint of this study is to compare 30-day survival outcomes between the icECMO and dcECMO approaches in RPCS patients. Secondary endpoints include the evaluation of adverse events and the identification of predictors that influence 30-day mortality.

Results

The study included 60 patients, 25 received icECMO and 35 treated with dcECMO due to RPCS. The icECMO group demonstrated significantly better 30-day survival rates (icECMO; 10 [40%] vs. dcECMO; 5 [14.3%], log-rank test; p=0.042). Despite comparable ECMO flow rate and ECMO RPM (rotations per minute) in the first day between the study groups ([icECMO; 4.5 l/min vs. dcECMO; 4.6 l/min, p=0.124], [icECMO; 3510 rpm vs. dcECMO; 3800 rpm, p=0.115], respectively), lactate levels were significantly higher in the dcECMO group on the 1st and 3rd post-extracorporeal life support (ECLS) days (p=0.006 and p=0.008, respectively). Gastrointestinal ischemia was more common in the dcECMO group (p=0.036). Successful ECMO weaning was more frequent in the icECMO group (56% vs. 22.9%, p=0.014). Multivariable logistic regression identified arterial lactate on the first day with a cutoff 4 mmol/l as an independent risk factor for 30-day mortality with Exp(B) of 8.9 (p=0.007).

Conclusions

Our findings suggest a potential survival advantage with the icECMO technique in patients undergoing central ECMO cannulation after RPCS. However, larger prospective studies are essential to confirm this observation.

## Introduction

Refractory postcardiotomy cardiogenic shock (RPCS) is a challenging complication that occurs after cardiac surgery, with an incidence ranging from 0.5% to 1.5% [1]. Veno-arterial extracorporeal membrane oxygenation (VA-ECMO) has become a standardized approach for managing RPCS over the years [2].

For implantation of VA-ECMO in these patients, generally, two primary methods are used: central cannulation of the right atrium and ascending aorta, or peripheral cannulation through the femoral vein and femoral or subclavian artery. Both approaches have been extensively discussed and compared in multiple studies [2-6].

The utilization of central ECMO offers several advantages, such as the use of previously established cardiopulmonary bypass (CBP) cannulas for antegrade flow. The larger size of these cannulas allows for better drainage from the right atrium, thereby enhancing a higher ECMO flow in the ascending aorta and reducing the risk of Harlequin syndrome. However, there are also notable disadvantages, including the need to maintain an open sternum and increased risks of bleeding, infection, and thromboembolic complications [6].

Direct central cannulation for ECMO (dcECMO) is usually achieved via an open sternotomy, direct atrio-aortic approach with provisional thoracic closure. Alternatively, indirect cannulation of the aorta via a vascular prosthesis (chimney) and venous drainage via the femoral vein as indirect central cannulation (icECMO) enables the immediate closure of the sternotomy after the index surgery. This can mitigate the above-mentioned complications, enhance early mobilization, and facilitate weaning from mechanical ventilation or extracorporeal life support (ECLS). At the same time, it also eliminates the need for surgical explantation of ECLS, and thus it should be considered as an approach of interest for central ECLS [3, 7].

Despite limited scientific evidence, we hypothesized that indirect central ECMO (icECMO) might lead to better patient outcomes compared to direct central ECMO (dcECMO) for patients with RPCS. To evaluate this hypothesis, we performed a retrospective analysis comparing icECMO and dcECMO approaches at a tertiary ECMO center. Our analysis primarily focused on 30-day survival rates, secondarily on postoperative complication rates, and on predictors of 30-day mortality.

## Materials and methods

Study population and groups

**Figure 1:**
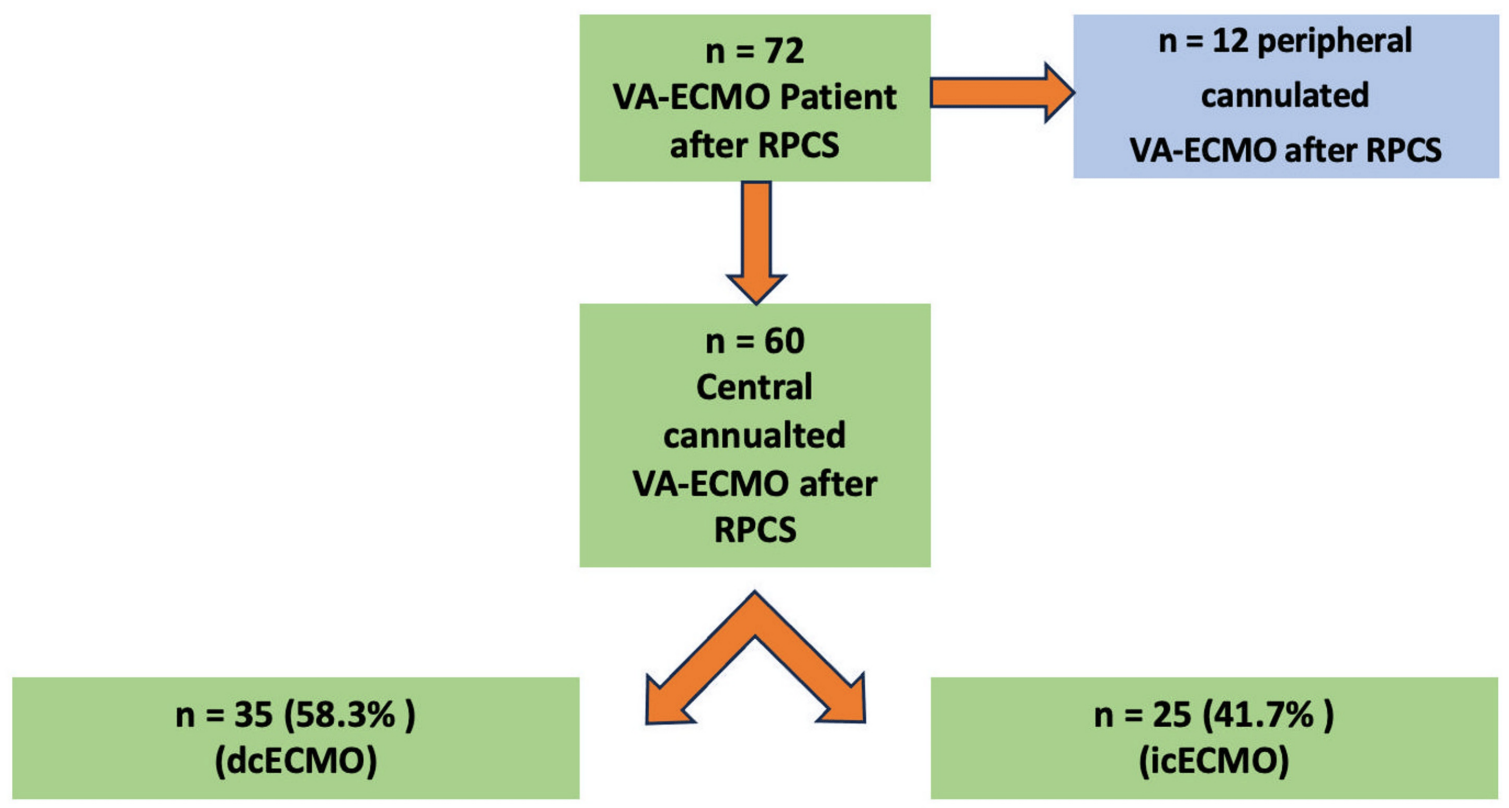
Flowchart depicting the patient selection process ECMO, extracorporeal membrane oxygenation; VA-ECMO, Venoarterial ECMO; RPCS, Refractory postcardiotomy cardiogenic shock; dcECMO, direct central cannulated ECMO; icECMO, indirect central cannulated ECMO.

This retrospective single-center study was approved by the ethical committee of the European Medical School Oldenburg-Groningen. We identified 72 adult patients (age >18-years) treated with ECMO therapy upon RPCS at our institution from January 2019 to December 2020. All patients with peripheral cannulated ECMO were excluded, thereby leaving 60 eligible patients. These patients were classified according to the method of central arterial cannulation. 35 and 25 patients were treated by dcECMO and icECMO, respectively (Figure 1). We analyzed the difference between these two groups in regard to preoperative, intraoperative, and postoperative variables. Furthermore, we employed here Kaplan-Meier survival curves for comparison of the 30-day survival

Data collection

The clinical characteristics of patients were retrospectively analyzed. Data collection included the evaluation of patient characteristics, cardiac risk factors, detailed surgery information, preoperative risk scores, laboratory parameters, complications, and clinical outcome, based on electronic patient records.

Cannulation techniques, ECMO protocol

For most patients that received ECLS due to failed weaning from CPB, we used the pre-existing cannulas in the ascending aorta as arterial (22 or 24 Fr. EOPA, Medtronic, Minneapolis, USA), and the right atrium, as a venous access. After establishing extracorporeal circulation via the ECLS, we performed either a patch implantation with a delayed chest closure or a temporary skin closure.

For some patients, we used the "chimney" technique for aortic cannulation. This involved the end-to-side anastomosis of an 8- or 10-mm vascular graft (Gelweave, Vascutek, Renfrewshire, Scotland, UK) to the ascending aorta, which was then tunneled through a subxyphoid skin incision on the frontal abdominal wall [7]. The arterial cannula was then connected subcutaneously to the vascular graft and secured in place using cable ties, while the venous cannula was inserted percutaneously through a femoral vein to initiate extracorporeal circulation. This approach enables the immediate closure of the chest after the index surgery.

If necessary, left ventricular drainage was achieved either through the insertion of a vent into the left ventricle via the right superior pulmonary vein and connected to the venous cannula, or by implantation of an Impella microaxial flow pump. A subgroup of patients underwent an intra-aortic balloon pump (IABP) implantation.

In our institution the main indication for VA-ECMO was RPCS. This was characterized by the inability to wean from CPB despite differentiated catecholamine treatment [8, 9]. Furthermore, secondary indications for the initiation of VA-ECMO were persistent malignant ventricular arrhythmia or cardiac arrest. The decision to initiate ECMO therapy was determined by the individual surgeon, which involved a combination of echocardiographic evaluation and clinical assessment to estimate the condition of patients before implementing ECLS [4]. Clinical assessment included low systemic blood pressure (<90 mmHg), low urine output, low SVO2 (<60 %), high lactate (> 4.0 mmol/l), and hemodynamic monitoring parameters using Swan-Ganz catheter [4, 10]. There were no specific criteria for the choice of cannulation approach, the decision was made at the discretion of the individual surgeon.

ECMO circuits consisted of a centrifugal pump (Rotaflow or Cardiohelp, Maquet, Rastatt, Germany), the membrane oxygenator (Quadrox, Maquet), and a circuit system (permanent life support system [PLS] or heart-lung support [HLS], Maquet). The institution's protocol for anticoagulation, monitoring, and weaning during ECMO treatment was identical to the previously described recommendations and guidelines [4, 10, 11, 12]. 

Briefly, right after the implantation of ECMO, anticoagulation treatment was initiated using heparin to achieve an activated clotting time of (ACT 180-200s). For patients who were stable, the dosage of heparin was adjusted based on their activated partial thromboplastin time with a target range of (aPTT 60-80s). In cases where patients were experiencing bleeding, the use of heparin was ceased immediately.

The clinical monitoring protocol included invasive arterial blood pressure monitoring via the radial artery to assess pulse pressure and compare oxygen saturation between the ECMO circuit and end organs. Pulse oximetry was crucial for evaluating peripheral oxygenation. A Swan-Ganz catheter was used to monitor left-sided filling pressures and provide continuous measurements of cardiac output and hemodynamics. Daily electrocardiography and echocardiography were performed to assess cardiac function. Continuous near-infrared spectroscopy (NIRS) was used to monitor tissue oxygenation between the brain and limbs [10].

ECMO weaning was systematically achieved following a comprehensive clinical evaluation, which included criteria such as maintaining sufficient urine output, and a low lactate concentration (< 2.2 mmol/l), ensuring a mean arterial pressure > 65 mmHg, minimal use of vasoactive and inotropic support to keep pulse pressure above 10 mmHg and maintain a left ventricular ejection fraction (LVEF) greater than 30%. Patients meeting these criteria, even with a decreasing ECMO flow rate to 2.0 L/min, were scheduled for ECMO explanation. This was achieved either in the intensive care unit for the icECMO-Patients or in the operating room for patient with dcECMO. We defined successful weaning as survival after explanation for over 24 h.

Endpoints of the study

The primary endpoint of this investigation was to evaluate 30-day survival rates. Secondary endpoints aimed to assess adverse events, various postoperative characteristics, and identify predictors influencing 30-day mortality during the index hospitalization. Patients who were either discharged or remained hospitalized throughout the observation period were classified as survivors.

Statistical analysis

Data was analyzed using SPSS Statistics version 29 (IBM Corp., Armonk, USA). Dichotomous variables were presented as total numbers (percentage), whereas continuous variables were checked for normality using the Shapiro-Wilk test and histogram inspection. Normally distributed continuous variables were expressed as the mean ± SD, while non-normally distributed continuous variables were reported as the median (IQR). Univariate analysis was performed using either Student t-test or Mann-Whitney U test for normally and non-normally distributed continuous variables, respectively. Comparison of categorical data was carried out using Pearson's chi square or Fisher exact tests, depending on the minimum expected count in each cross-tab. The statistical significance was set at P < 0.05.

Kaplan-Meier curves were used to illustrate survival differences among study groups within the first 30 days after VA-ECMO initiation. The log-rank test was applied for comparing the curves. Additionally, we performed univariable and multivariable logistic regression analyses. Only variables that were clinically relevant to the outcome and showed significant differences (p < 0.05) between the icECMO and dcECMO groups after ECMO initiation, such as lactate levels and gastrointestinal ischemia, were included in the univariable analysis. Significant variables (p < 0.05) from the univariable analysis were then included in a multivariable logistic regression model to examine their impact on 30-day mortality after VA-ECMO implantation. Results are reported as Exp(B) with 95% confidence intervals (CIs).

## Results

Baseline characteristics

The study included 60 eligible patients meeting the inclusion criteria. Out of these patients, 25 (41.7 %) received indirect central cannulation with a prosthesis (icECMO), while 35 (58.3 %) were treated using direct central cannulation without a prosthesis (dcECMO). Table 1 presents the patient characteristics and demographics. Notably, the baseline characteristics were equally distributed, resulting in no statistically significant differences between the two groups.

**Table 1:** Preoperative characteristics IQR, interquartile range; SD, Standard deviation; LVEF, left ventricular ejection fraction; CKD, chronic kidney disease; COPD, chronic obstructive pulmonary disease; MI, myocardial infarction; ECMO, extracorporeal membrane oxygenation; dcECMO, direct central cannulated ECMO, icECMO, indirect central cannulated ECMO.

Variable	icECMO n (%)	dcECMO n (%)	P-value
Age, years, mean ± SD	68.6 ± 10.2	64.9 ± 13.7	0.431
Sex, male	18 (72)	26 (74.3)	1.000
BMI, kg/m^2^, mean ± SD	28.2 ± 5	30.1 ± 5.7	0.810
Hypertension	15 (60)	21 (60)	1.000
Diabetes mellitus typ I	4 (16.0)	6 (17.1)	1.000
Dyslipidemia	10 (40)	12 (34.3)	0.787
History of smoking	14 (56)	15 (42.9)	0.433
Peripheral arterial disease	6 (24)	6 (17.1)	0.532
Previous MI within 90 days	9 (36)	10 (28.6)	0.583
Previous Sternotomy	4 (16)	7 (20)	0.748
Atrial fibrillation	11 (44)	13 (37.1)	0.606
Previous Stroke	4 (16)	6 (17.1)	1.000
LVEF, %, (Median,IQR)	40 (30-50)	45 (35-60)	0.094
LVEF < 35%	7 (28)	6 (17.1)	0.335
CKD	13 (52)	15 (42.9)	0.601
COPD	8 (32)	4 (11.4)	0.099
Active endocarditis	4 (16)	9 (25.7)	0.527
Critical preoperative state	3 (12)	3 (8.6)	0.686
Euroscore II, (Median,IQR)	7.38 (3.84-16.06)	6.75 (2.93-14.53)	0.753

Intraoperative and ECMO results

Table 2 demonstrates the intraoperative data, such as the type of cardiac procedure before applying ECLS due to RPCS. Coronary artery bypass grafting (CABG), as an isolated procedure, was the leading procedure with 10 patients (40%) in the icECMO group and with 11 patients (31.4%) in the dcECMO group. It is noteworthy that icECMO (chimney technique) was not utilized in any case of the thoracic aorta surgery. However, no statistically significant difference was observed between the study groups (p=0.506).

**Table 2:** Intraopertive results CABG, coronary artery bypass grafting; AVR, aortic valve replacement; MVR/r, mitral valve replacement/reconstruction; TVR/r, tricuspidal valve replacement/reconstruction; ACC, Aortic cross-clamp; CPB, Cardiopulmonary bypass; LV-venting, left ventricular venting; IABP, intra-aortic balloon pump; RPCS, refractory postcardiotomy shock; RPM, rotations per minute; ECMO, extracorporeal membrane oxygenation; dcECMO, direct central cannulated ECMO; icECMO, indirect central cannulated ECMO.

Variable	icECMO n (%)		dcECMO n (%)	P-value
Type of cardiac procedure				
Isolated CABG	10 (40)		11 (31.4)	0.587
CABG + AVR	5 (20)		4 (11.4)	0.470
Isolated AVR	4 (16)		4 (11.4)	0.708
Isolated MVR/r	1 (4)		2 (5.7)	1.000
±CABG ± AVR ±MVR/r± TVR/r	3 (12)		6 (17.1)	0.722
Thoracic aorta procedures	0 (0)		2 (5.7)	0.506
Others	2 (8)		6 (17.1)	0.449
ACC time, min, (Median,IQR)	56.0 (37.0-108.0)		65.0 (51.0-106.0)	0.401
CPB time, min, (Median,IQR)	128.0 (78.5-171.5)		144.0 (103.0-192.0)	0.098
Impella	3 (12)		3 (8.6)	0.686
LV-venting	2 (8)		4 (11.4)	1.000
IABP	10 (40)		7 (20)	0.145
RPCS introperative	16 (64)		23 (65.7)	1.000
RPCS in ICU	9 (36)		12 (34.3)	1.000
Chest left Open intraoperatively	2(8)		35 (100)	<0.001
ECMO characteristics on 1^st^ day and Inflow canula sizes				
22 Fr. n (%)	5 (20)		11 (31.4)	0.386
24 Fr. n (%)	20 (80)		24 (68.6)	0.386
ECMO flow rate, (Mean±SD)	4.5 ± 0.77		4.6 ± 0.64	0.124
ECMO RPM, (Median, IQR)	3510 (2990-3832)		3800 (3300-4000)	0.115

The duration of aortic cross-clamp (ACC) and cardiopulmonary bypass (CPB) was comparable in the two study groups (56 min vs. 65 min, p= 0.401 and 128 min vs. 144.0 min, p= 0.098, respectively). Implantation of Impella was performed on three (12%) icECMO patients and on three (8.6%) of dcECMO patients, while two patients (8%) of icECMO group and four (11.4%) of dcECMO group underwent left ventricular venting through insertion of a canula in the right superior pulmonary vein. In 17 patients of both groups an IABP was implanted transfemorally. No significant differences were observed between the two groups in terms of IABP or Impella implantation, or left ventricular venting (LV-venting). The majority of patients in the two study groups (icECMO; 16 [64 %], dcECMO; 23 [65.7%]) received the VA-ECMO because of inability to wean from the cardiopulmonary bypass in the operating room. 36% of icECMO patients and 34.3% of dcECMO patients received VA-ECMO on the ICU due to delayed RPCS, ventricular arrhythmia or cardiac arrest. The chest was left open following ECMO initiation for all dcECMO patients, while the chest was closed in all icECMO patients except for two patients, who treated with icECMO and LV-venting in the operation room.

The most commonly used ECMO inflow canula size for both groups was the 24 Fr. canula (icECMO; 20 [80 %], dcECMO; 24 [68.6%], p=0.386). The mean ECMO flow rate in the first day and the median ECMO RPM (rotations per minute) were comparable in the study groups ([icECMO; 4.5 l/min vs. dcECMO; 4.6 l/min, p=0.124], [icECMO; 3510 rpm vs. dcECMO; 3800 rpm, p=0.115], respectively).

Table 3 summarizes the laboratory parameters measured before and during ECMO therapy. The data indicate that there were no significant differences between the groups, except for lactate levels. Notably, the lactate levels were similar in the pre-ECLS phase, but significantly higher in the dcECMO group on the 1st and 3rd post-ECLS day (p=0.006, p=0.008, respectively). However, the median lactate level on the 3rd post-ECLS day fall within the normal reference range of 0.5-2.2 mmol/L in both study groups.

**Table 3:** Laboratory findings IQR, interquartile range; ECLS, Extracorporeal life support; ECMO, extracorporeal membrane oxygenation; dcECMO, direct central cannulated ECMO; icECMO, indirect central cannulated ECMO. *the highest lactate level just before ECMO-Initiation.

Variable	icECMO (Median, IQR)	dcECMO (Median, IQR)	P-value
Pre ECLS			
Lactate, mmol/L*	4.1 (2.8-6.5)	3.8 (2.4-7.6)	0.816
Creatinine, mg/dL	1.2 (0.9-1.7)	1.1 (0.9-1.5)	0.235
Total bilirubin mg/dL	0.75 (0.5-1.1)	0.7 (0.5-1.4)	0.824
1^st^ post ECLS day			
Lactate, mmol/L	2.1 (1.6-4.0)	4.5 (2.7-6.2)	0.006
Creatinine, mg/dL	1.6 (1.1-1.8)	1.4 (1.0-1.6)	0.232
Total bilirubin mg/dL	1.8 (1.4-3.1)	1.8 (1.0-3.5)	0.976
3^rd^ post ECLS day			
Lactate, mmol/L	0.9 (0.9-1.5)	1.2 (1.2-2.5)	0.008
Creatinine, mg/dL	1.6 (0.9-2.4)	1.2 (1.0-1.7)	0.119
Total bilirubin mg/dL	1.7 (1.0-4.2)	3.2 (1.2-5.5)	0.205
5^th^ post ECLS day			
Lactate, mmol/L	1.2 (1.1-1.6)	1.4 (1.1-1.8)	0.477
Creatinine, mg/dL	1.2 (0.8-2.3)	1.2 (0.9-1.6)	0.483
Total bilirubin mg/dL	2.1 (0.7-5.6)	3.8 (1.7-6.5)	0.178

Early outcomes after ECMO initiation

Table 4 illustrates the early outcomes following ECMO implantation for the study groups. Although the complication rate was comparable between both groups, it was evident that the dcECMO group trended towards a higher incidence of gastrointestinal complications (p=0.067). Specifically, there was a greater occurrence of gastrointestinal ischemia (icECMO, 0 [0 %] vs. dcECMO, 6 [17 %], p=0.036).

**Table 4:** Clinical complication RBC, red blood cells; ECMO, extracorporeal membrane oxygenation; dcECMO, direct central cannulated ECMO; icECMO, indirect central cannulated ECMO. * in the first 24 hours after ECMO initiation.

Variable	icECMO n (%)	dcECMO n (%)	P-value
Major bleeding need to exploration	12 (48)	22 (62.9)	0.298
Cannula site bleeding	1 (4)	5 (14.3)	0.386
Surgical site bleeding	8 (32)	15 (42.9)	0.432
RBC transfusion*, units, (median, IQR)	5 (2.2-7)	4 (3-8)	0.739
Chest tube outcome*, ml, (median, IQR)	750 (412-3430)	700 (475-1850)	0.765
Gastrointestinal complication	1 (4)	8 (22.9)	0.067
Gastrointestinal bleeding	1 (4)	4 (11.4)	0.390
Gastrointestinal ischemia	0 (0)	6 (17.1)	0.036
Limb ischemia	0 (0)	1 (2.9)	1.000
Neurological dysfunction	5 (20)	11 (31.4)	0.386
Hypoxic-Ischemic Encephalopathy	2 (8)	3 (8.6)	1.000
Intracerebral hemorrhage	1 (4)	1 (2.9)	1.000
Ischemic stroke	3 (12)	8 (22.9)	0.332
Acute renal dysfunction needs to Dialysis	13 (52)	23 (65.7)	0.301
Respiratory dysfunction	13 (52)	12 (34)	0.194
Tracheostomy	9 (36)	7 (20)	0.238
Reintubation	7 (28)	4 (11.7)	0.174
Hepatic dysfunction	13 (52)	25 (73.5)	0.106
Infection	13 (52)	17 (48.6)	1.000
Pneumonia	9 (36)	13 (37.1)	1.000
Sepsis	3 (12)	11 (31.4)	0.122

**Figure 2:**
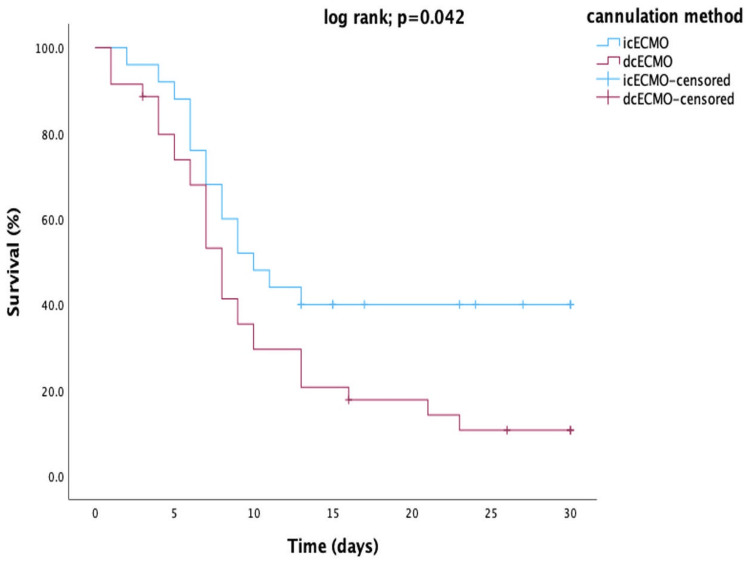
30-day survival for central VA-ECMO patients based on central cannulation method (icECMO vs. dcECMO). Patients who were discharged or transferred to a rehabilitation facility within 30 days were classified as censored. ECMO, extracorporeal membrane oxygenation; dcECMO, direct central cannulated ECMO; icECMO, indirect central cannulated ECMO; VA-ECMO, venoarterial ECMO.

VA-ECMO duration and hospital stay did not show statistically significant differences between both groups (Table 5). Nonetheless, there was a significant difference in the ability of successful ECMO weaning, favoring the icECMO group (icECMO; 14 [56 %] vs. dcECMO; 8 [22.9 %], p=0.014). However, the Kaplan-Meier survival curve showed a significant difference in the 30-day survival outcomes in favor of the icECMO group (icECMO; 10 [40%] vs. dcECMO; 5 [14.3%], log-rank test; p=0.042; Figure 2).

**Table 5:** ECLS outcomes ECLS, extracorporeal life support; VA-ECMO, venoarterial extracorporeal membrane oxygenation; LVAD, left ventricle assist device; ECMO, extracorporeal membrane oxygenation; dcECMO, direct central cannulated ECMO; icECMO, indirect central cannulated ECMO.

Variable	icECMO n (%)	dcECMO n (%)	P-value
VA-ECMO Duration, d, (median, IQR)	6 (4-8)	7 (5-9)	0.386
Successful ECMO-weaning	14 (56)	8 (22.9)	0.014
LVAD implantation	1 (4)	1 (2.9)	1.000
Hospital stay, d, (median, IQR)	10 (7-15)	8 (5-13)	0.125
30-day Survival	10 (40)	5 (14.3)	0.035

After performing both univariable and multivariable logistic regression analyses, we identified an independent factor for 30-day mortality. Arterial lactate on the first day, with a cutoff value of 4 mmol/l, emerged as an independent risk factor for 30-day mortality with an Exp(B) of 8.9 (p=0.007) (Table 6).

**Table 6:** Variables exhibiting statistically significant differences between study groups (icECMO vs. dcECMO) and their influence on 30-day mortality after central VA-ECMO initiation. Exp(B), exponentiation of the B coefficient; CI, confidence interval; ECMO, extracorporeal membrane oxygenation; dcECMO, direct central cannulated ECMO; icECMO, indirect central cannulated ECMO; VA-ECMO, venoarterial ECMO.

Univariable logistic regression	Exp(B) (95% CI)	P-value
Lactate preoperative	1.1 (0.9-1.3)	0.270
Lactate 1^st^ day	1.9 (1.2-3.0)	0.006
Lactate 3^rd^ day	2.8 (0.9-8.9)	0.078
Lactate over 4 mmol/L in the 1^st^ day	8.9 (1.8-44)	0.007
Lactate over 3 mmol/L in the 3^rd^ day	3.6 (0.4-31)	0.224
Gastrointestinal ischemia	1.7 (0.18-16)	0.623
Multivariable logistic regression	Exp(B) (95% CI)	P-value
Lactate over 4 mmol/L in the 1^st^ day	8.9 (1.8-44)	0.007

## Discussion

Refractory postcardiotomy cardiogenic shock is a critical postoperative complication following cardiac surgery and associated with an increased mortality and morbidity when compared to any other underlying cause of cardiogenic shock [13].

VA-ECMO has emerged as the predominant approach in managing this fatal complication among patients with RPCS [14]. While the outcomes of VA-ECMO in RPCS have been intensively discussed [1, 7, 8, 13, 15], there is currently no universally accepted concept for VA-ECMO access in RPCS patients. Variations exist in central or peripheral cannulation, and whether peripheral cannulation is achieved via the subclavian or femoral artery [2-4, 6, 16]. Notably, central cannulation is the more frequently used strategy for initiating ECLS in RPCS patients [1, 9].

However, due to the open chest situation in the operating room, central cannulation is often the preferred choice in RPCS patients as direct access to the aorta is readily available, and thereby faster than peripheral cannulation [5, 14]. In our institution, central arterial cannulation was the most common method for implantation of VA-ECMO for all RPCS-patients, either in the operating room or after admission to the ICU (cECMO n=60 vs. pECMO n=12, Figure 1). In this context, our study focused exclusively on central cannulation. We aimed to determine which of its two modalities, icECMO and dcECMO, is associated with a better outcome for RPCS patients.

Our single-center data showed a 30-day survival rate of 25%, which is similar to the recently published data. In a systematic review, Mariscalco et al. reported a 30-day survival rate of approximately 28% for patients with central VA-ECMO due to RPCS [17]. A large cohort study conducted by Rastan et al. showed similar results with an overall 30-day survival rate of 24.8% in 517 patients treated with VA-ECMO after RPCS. Still, the authors did not account for potential variations in survival outcomes based on different central cannulation methods employed during the procedure [1]. However, in our study, the comparison of 30-day survival rate between the two central cannulation strategies yielded a significantly better survival outcome favoring the icECMO group (icECMO; 10 [40%] vs. dcECMO; 5 [14.3%], log rank; p=0.042; Figure 2). This result from our relatively small patient cohort suggests that the icECMO technique may offer more effective support for patients undergoing central ECMO due to RPCS and potentially leading to improved survival outcomes.

It is noteworthy that on the first day, the mean ECMO flow rate between the two groups was almost identical (icECMO; 4.5 l/min vs. dcECMO; 4.6 l/min), indicating that both techniques provided a similar level of initial hemodynamic support (p=0.124). However, the median ECMO RPM was slightly higher in the dcECMO group without a significant difference (icECMO; 3510 rpm vs. dcECMO; 3800 rpm; p=0.115). This might reflect a need for more vigorous pump activity to maintain the same flow rate, potentially indicating higher resistance or less efficient flow dynamics in the dcECMO setup. Notably, the most commonly used inflow cannula size was 24 Fr. for both groups. However, the 24 Fr. inflow cannula in the dcECMO group was connected directly to the aorta with a diameter of 8 mm. In contrast, the same inflow cannula in the icECMO group was connected to the aorta through a prosthesis with a diameter of 10 mm. Similarly, the diameter of the 22 Fr. inflow cannula at the aorta in the dcECMO group was 7.3 mm. Conversely, in the icECMO group, the same inflow cannula was connected to the aorta through a prosthesis with a diameter of 8 mm. This difference in the diameter at the aorta could imply that icECMO achieves the necessary flow rates more efficiently.

Lactate is a metabolic product of anaerobic glycolysis, indicating insufficient oxygen delivery. It is suggested as a marker for tissue perfusion, affected by both microcirculation and microcirculation [18]. Hyperlactatemia is associated with an increased mortality rates in patients undergoing cardiac surgery and particularly during ECMO support [19-21]. The lactate levels before ECMO initiation was high but comparable between the both study groups (p=0.816). Unlike studies reported by Rastan, Fux, and Hamiko et al. [1, 18, 22], our findings align with report by Slottosch et al. [21] indicating that the pre-ECLS lactate value did not exhibit a significant association with 30-day mortality.

Interestingly, the median lactate level on the first day in the icECMO (chimney) group tended to fall within the normal reference range of 0.5-2.2 mmol/L. In contrast, the dcECMO group exhibited a persistently elevated median lactate level of 4.5 mmol/L (p=0.006). The persistently elevated lactate levels within 24 hours after ECMO initiation emerged as a robust predictor for 30-day mortality (p=0.006), aligning with other published data [1, 21]. Of note, two studies reported lactate serum concentration > 8 mmol/L 24 hours after ECMO-initiation as a potential negative predictor for survival [23, 24]. In order to further refine our findings, we then established a cutoff value of 4 mmol/l for lactate in the first 24 hours. Lactate values over this threshold were identified as an independent risk factor for 30-day mortality, with Exp(B) of 8.9 (p=0.007). Generally, icECMO technique showed lower lactate levels, suggesting adequate tissue perfusion and sufficient oxygen delivery, when compared to the dcECMO.

In our cohort, 34 (56.6%) patients required reexploration due to various bleeding complications (e.g., mediastinal bleeding, tamponade, or bleeding at the cannula/graft site). We initially anticipated a higher incidence of reexploration because of bleeding complications in the dcECMO group due to the increased risk of bleeding from the sternum and soft tissue, as already discussed in the literature [3-5]. Conistent with our expectations, the need for reexploration due to bleeding was more frequent in the dcECMO group (icECMO; 12 [48 %], dcECMO; 22 [62.9%]), but this difference was not statistically significant (p=0.298). However, the need for RBC transfusion and the chest tube outcomes were comparable between the two groups (p=0.739, p=0.765, respectively, Table 4). This suggests, that whether the thorax is already closed or still open does not significantly increase the risk for thoracic reexploration, RBC transfusion, or chest tube outcomes.

Although no significant differences were documented in respiratory, neurologic, vascular, hepatic, renal complication and infection rates between the two cannulation strategies, it is important to note that the dcECMO group exhibited a higher incidence of gastrointestinal ischemia (p=0.036). Gastrointestinal ischemia contributes to increased lactate levels, as already dicussed in the literature [25-26]. The pathophysiology of gastrointestinal ischemia involves hypoperfusion in mesenteric blood flow [26], which can consequently serve as an indicator of insufficient blood flow to the end organ in the dcECMO group.

The successful weaning rate from VA-ECMO was significantly higher in favor of the icECMO group compared to the dcECMO group (icECMO; 56 % vs. dcECMO; 22.9 %, p=0.014). We assume that the improved outcome in the icECMO group was associated with the initial sternal closure after the index surgery, thereby resulting in a faster return to the usual anatomical situation and physiological function of the heart and lungs. This step appeared to play a significant role in reducing the duration of postoperative anesthesia and mitigating its related complications. ECMO explantation can differ depending on the chosen cannulation approach. In the icECMO (chimney) group, the arterial cannula of ECMO is located in the vascular graft at the subxiphoid exit, making the explantation relatively straightforward. This procedure takes place in the intensive care unit under local anesthesia, eliminating the need for surgical explantation of VA-ECMO in the operating room. In contrast, the dcECMO group underwent a prolonged period of anesthesia until cardiac recovery was achieved. Their ECMO explantation involved the invasive removal of both arterial and venous cannulas from the chest cavity. In summary, icECMO patients experienced less invasive procedures, reduced physiological stress and faster recovery along with the higher rate of successful weaning from ECMO.

Limitations

This study has several limitations, including its retrospective, nonrandomized design, single-center setting, the individual surgeon's choice of surgical approach, and the short-term follow-up. The small sample size may have diminished statistical power, potentially increasing selection bias and affecting the results. Moreover, the study did not explore how variations in surgeon experience or evolving practices over time might have influenced the outcomes. To address these issues, future research should include prospective randomized multicenter trials to better define indications and cannulation strategies for central ECLS therapy.

## Conclusions

Our retrospective single-center study offers valuable insights into the potential benefits of icECMO approach in the management of RPCS. It demonstrates improved survival rates, significantly lower lactate levels, reduced rates of gastrointestinal ischemia, and a higher rate in weaning. However, these findings should be interpreted with caution due to the limited patient population. prospective randomized trials are essential to confirm this preliminary observation.
